# Time in Redox Adaptation Processes: From Evolution to Hormesis

**DOI:** 10.3390/ijms17101649

**Published:** 2016-09-29

**Authors:** Mireille M. J. P. E. Sthijns, Antje R. Weseler, Aalt Bast, Guido R. M. M. Haenen

**Affiliations:** Department of Pharmacology and Toxicology, P.O. Box 616, Maastricht University, 6200 MD Maastricht, The Netherlands; a.weseler@maastrichtuniversity.nl (A.R.W.); a.bast@maastrichtuniversity.nl (A.B.); g.haenen@maastrichtuniversity.nl (G.R.M.M.H.)

**Keywords:** hormesis, time, redox adaptation, glutathione, acrolein, flavonoids

## Abstract

Life on Earth has to adapt to the ever changing environment. For example, due to introduction of oxygen in the atmosphere, an antioxidant network evolved to cope with the exposure to oxygen. The adaptive mechanisms of the antioxidant network, specifically the glutathione (GSH) system, are reviewed with a special focus on the time. The quickest adaptive response to oxidative stress is direct enzyme modification, increasing the GSH levels or activating the GSH-dependent protective enzymes. After several hours, a hormetic response is seen at the transcriptional level by up-regulating Nrf2-mediated expression of enzymes involved in GSH synthesis. In the long run, adaptations occur at the epigenetic and genomic level; for example, the ability to synthesize GSH by phototrophic bacteria. Apparently, in an adaptive hormetic response not only the dose or the compound, but also time, should be considered. This is essential for targeted interventions aimed to prevent diseases by successfully coping with changes in the environment e.g., oxidative stress.

## 1. Adaptation or Hormesis in General

The only constant in the atmosphere on Earth is that its composition was, is, and will never be constant over time. As a consequence, life on Earth needs to continuously adapt to an ever-changing environment. One of the major changes was the introduction of oxygen (O_2_) in the Earth’s atmosphere, roughly 2.5 billion years ago, to a level of 21 percent nowadays [1]. 

The chemical reactivity of oxygen created lifeforms that acquire their energy from oxidation of compounds. In humans, the supply of, especially, carbohydrates and fats creates a redox potential that fuels all reactions in our body. Eating and exercise will cause the redox potential to gradually fluctuate in time. A sudden block of blood supply drops the oxygen tension and shows that the redox potential can also change drastically within seconds. 

If the level of oxygen is too low, then reductive stress is experienced. The low oxygen level results in e.g., an impaired mitochondrial respiratory chain function. Alternatively, when the level of oxygen is too high, oxidative stress will be the result because of an increase in reactive oxygen species (ROS) due to an extensive use of the aerobic mitochondrial respiratory chain. These ROS may, subsequently, lead to cell death and can destroy vital cell structures, such as membranes, proteins, and DNA. This illustrates the oxygen paradox, which implies that in addition to the dependency of aerobic life on the chemical reactivity of oxygen, the chemical reactivity of oxygen also results in the destruction of vital cell structures. The latter process, known as aging, will finally kill the organism [2,3]. 

To mitigate oxygen toxicity and maintain the redox potential within narrow limits, cells are equipped with an intricate antioxidant network. The glutathione (GSH) system is a pivotal part of this network. Apart from antioxidants, there are compounds that can induce oxygen toxicity by promoting the formation of ROS. 

A peculiar paradox called the oxygen paradox is that adaptation against increased oxygen toxicity is due to oxidative changes in these cells. As will be outlined, this can be due to a low, subtoxic dose of oxidants that can induce changes on a relatively short time scale to antioxidant enzymes (that become more active after oxidation), to transcription factors that act as a redox sensor, and oxidative damage to DNA can lead, by chance, to mutated species that are better equipped to deal with the increased oxygen toxicity i.e., evolution [4].

**Figure 1:**
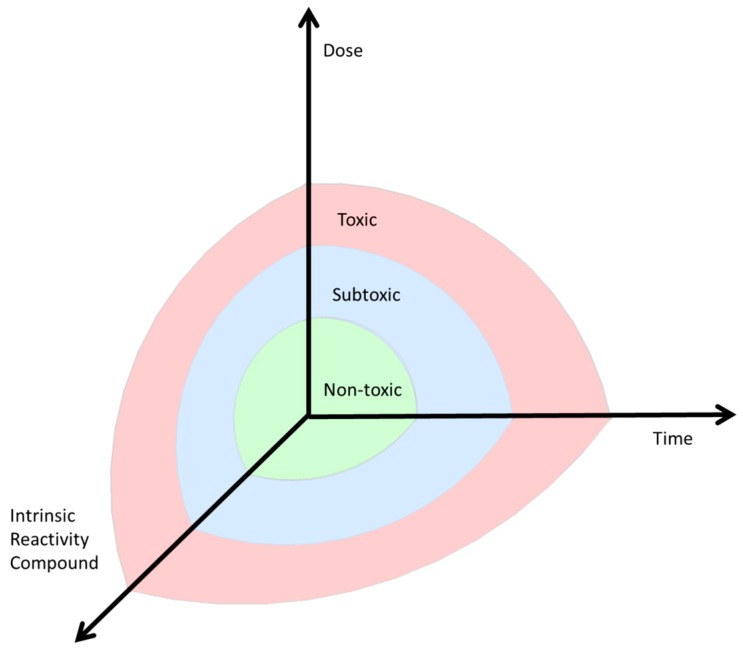
The dose, the compound involved, and the timing of exposure influence whether homeostasis exists (green = normal ≠ safe, but adapted to toxicity, as low as reasonably achievable (ALARA)), an adaptive response (blue, subtoxic) or toxicity (red, toxic) is induced.

A response in cells or organisms induced by a low, subtoxic dose of a compound that can induce changes in the environment is called hormesis [5]. Next to hormesis, other terms have also been suggested to describe this phenomenon. The historical development of these terms has been thoroughly described by Davies [6]. First, Claude Bernard introduced the concept of a constant internal environment in the body in 1865 [7]. This concept was named “homeostasis”. If this internal steady state is triggered by exogenous factors, a state of “heterostasis” may occur as defined by Hans Seyle in the 1970s [8]. Later, the term “allostasis” was used instead of “heterostasis”. Then, Southem and Ehrlich introduced the current term “hormesis” as an adaptive response inducing either repair mechanisms or enhanced protection after exposure to low doses of a compound [9]. This concept is currently still used and Calabrese introduced this concept in toxicological research [10]. Hormesis is involved in many physiological processes, but in this review we focus on the ability of cells to adapt to a changing redox potential, called redox regulation, which is involved in many different cellular processes. When the changes in redox potential are too drastic, these adaptive systems fail. This will damage organs and contribute to the development of e.g., cardiovascular diseases, cancer, and metabolic diseases. This means that health can be defined as the ability of a cell or organism to keep varying physiological processes within narrow limits (Figure 1). Ideally, formation and scavenging of ROS is well balanced, a condition known as ROS homeostasis. When this balance is disturbed, a specific cellular function can be triggered; for example, cell survival. Since these physiological adaptations can lead to a (transient) change or expansion of the internal steady state, Davies recently suggested the term “adaptive homeostasis” [6]. 

For the present review the ability of the antioxidant network to adapt to the environment will be illustrated by the GSH system with a special focus on the timing of the adaptive response. It should be noted that the adaptive mechanisms are emphasized (hormesis) and not the change in internal steady state (adaptive homeostasis). Relatively short-term adaptive mechanisms belong to the phenomenon called hormesis, whereas the development of adaptation on a time scale of months or years are not defined as hormesis, but can be seen as a kind of adaptation.

GSH acts as a reductant that can annihilate the chemical reactivity of ROS in a redox reaction. Various adaptive mechanisms of the GSH system have been developed to cope with the varying oxygen tension.

## 2. Short Term—Enzymatic Level

Sudden changes in the environment calls for a direct adaptation, which should develop within seconds or minutes. First, this can be achieved by modification of enzyme activity of one of the redox defense enzymes of the GSH system. This type of short-term adaptation is seen for the (microsomal) glutathione-*S*-transferases ((M)GSTs) [11]. They can protect by catalyzing the scavenging of ROS by GSH. ROS can regulate GST activity. The enzyme contains redox-sensitive sulfhydryl groups that readily react with ROS. Oxidation of sulfhydryl groups leads to activation. This indicates that the more ROS that are formed, the more sulfhydryl groups that are modified, and the higher the GST activity becomes, resulting in more protection [12]. This is a form of short-term redox adaptation by modifying a redox-sensitive group on the enzyme and thereby boosting its activity.

Secondly, short-term redox adaptation could also target the synthesis of GSH by regulating gamma-glutamylcysteine synthetase (γGCS), the rate-limiting enzyme of GSH synthesis. Γ-GCS consists of two subunits, one heavy catalytic subunit (γ-GCS-HS) of 73 kDa and one light regulatory subunit (γ-GCS-LS) of 30 kDa. The catalytic subunit has binding sites for the three substrates, γ-glutamic acid, cysteine, and adenosine triphosphate (ATP). The regulatory subunit contains a binding site for GSH and binding of GSH functions as a feedback inhibitor [13]. Under normal conditions, 80% of the available γGCS is inactive due to the reduction of a disulfide bond interaction between the regulatory and the catalytic subunit [13,14]. High oxygen tension and excessive ROS production will deplete GSH and restore the disulfide bond. This leads to a conformational change in the catalytic subunit, increasing the affinity of the catalytic subunit for its substrates and thereby stimulating GSH synthesis [15,16] (Table 1). Furthermore, via this direct enzymatic redox regulation, a short-term adaptation of the protective GSH system can be achieved. 

Third, adaptation can also be achieved by modification of the supply of enzymatic cofactors to ensure a regeneration of the cellular GSH pool. Scavenging of ROS results in the formation of glutathione disulfide (GSSG). For efficient protection GSSG needs to be converted back into GSH. In order to keep GSH levels high, glutathione reductase (GR) recycles GSH from GSSG at the expense of nicotinamide adenine dinucleotide phosphate (NADPH). To prevent depletion of NADPH an adaptive mechanism has evolved to promote NADPH formation during oxidative stress. During oxidative stress, glyceraldehyde-3-phosphate dehydrogenase (GAPDH), an essential enzyme in glycolysis, is inactivated by oxidation. This redirects the energy-generating process into the pentose phosphate pathway [17,18,19,20]. The switch from reduced nicotinamide adenine dinucleotide (NADH) production by GAPDH to NADPH production by the pentose phosphate pathway could also be seen as a form of short-term adaptation. This ensures that GSH levels are sufficient for thiol peroxidases to scavenge hydrogen peroxide (H_2_O_2_) and other ROS [17].

It should be noted that previous short-term adaptive mechanisms will induce changes that are beneficial on the level of an individual. Enzymatic, but also some transcriptional, changes contribute to the survival of a cell, an organism, or individual in the ever-changing environment. 

## 3. Long Term Adaptation—Transcriptional, Epigenetic, and Genomic Level

In addition to direct short-term effects of O_2_ levels, long term adaptive effects are also induced. For long-term adaptive effects a subdivision can be made in effects within hours to days and over generations.

### 3.1. Transcriptional Level

First, within hours to days, adaptation is mainly induced via altered gene transcription.

For example, ROS induce adaptation by up-regulating the endogenous antioxidant level within hours to days. ROS oxidize specific cysteines (Cys273, 288, 151) from Kelch like-ECH-associated protein 1 (Keap1), thereby increasing nuclear translocation of nuclear factor (erythroid-derived 2)-like 2 (Nrf2) and enhancing the transcription of antioxidant response element (ARE) containing antioxidant genes, including γGCS [21]. This subsequently contributes to an increased GSH synthesis and increased protection against oxygen toxicity over a longer term. GSH protects against ischemia-reperfusion damage by maintaining nitric oxide (NO)-induced vasodilation and regulating blood flow to several parts of the body [22]. Oxidative stress has been shown to impair NO-induced vasodilation of blood vessels [23]. However, *N*-acetylcysteine (thiol) supplementation improved vasodilation of arteries, indicating GSH protects the vasodilatory function of the vessels [24].

A low O_2_ tension also induces a long-term transcriptionally-mediated adaptive process via the hypoxia-inducible factor 1-α (HIF1α)-pathway. Prolyl hydroxylases are O_2_ sensitive. In the case of normoxia, prolyl hydrolysases bind O_2_ to their central ferrous iron moiety and use 2-oxoglutarate as a substrate to hydroxylate HIF1α on the oxygen-dependent degradation domain; thereby succinate and carbon dioxide are formed. Then the E3 ligase Von Hippau Lindau protein is able to bind HIF1α for subsequent ubiquitinylation and proteasomal degradation [25]. CBP/p300 coactivator interaction is prevented by hydroxylation of the carboxy-terminal chain of HIF1α by an oxygen-dependent asparaginyl hydroxylase [25]. However, during hypoxia or low oxygen tension, there is no oxygen present to keep the central iron of prolyl hydroxylase in its ferrous state [25]. HIF1α is not hydroxylated anymore, but contributes to an increase in oxygen tension over the long-term. HIF1α translocates to the nucleus and recruits its coactivators including CBP/p300. Together they bind to the hypoxia response elements and up-regulate gene expression of genes including vascular endothelial growth factor (VEGF) or inducible nitric oxide synthase (iNOS) that are involved in angiogenesis or new vessel generation [18]. Additionally, on an energetic level an adaptive response is induced, promoting anaerobic metabolism by up-regulating of gene expression of genes like GAPDH [26]. 

**Figure 2:**
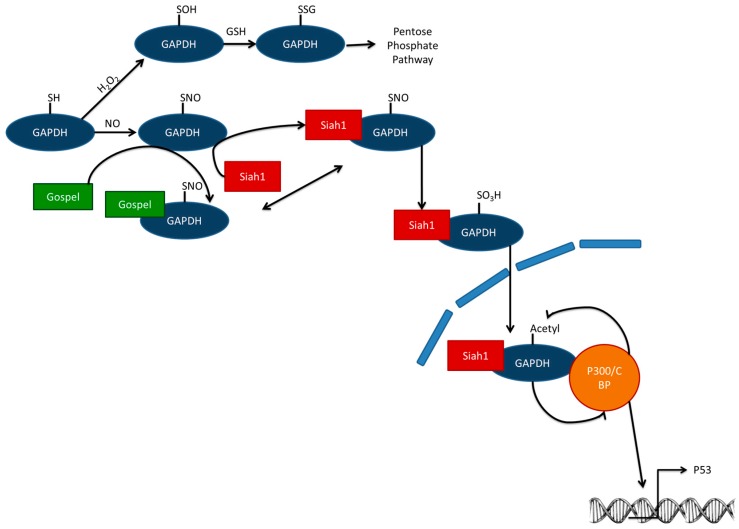
GAPDH can be oxidized and *S*-glutathionylated, which inhibits its glycolytic function and directs a metabolic switch towards the pentose phosphate pathway. If there is too much stress, not only is GOSPEL *S*-nitrosylated, but also GAPDH, which now prefers binding Siah1 instead of GOSPEL. Siah1 has a nuclear localization signal. In the nucleus, GAPDH complexes with the co-activator CREB-binding protein (CBP)/p300. CBP/p300 subsequently acetylates GAPDH. Acetylated GAPDH boosts the CBP/p300 acetylating function. This enhances transcription of GAPDH downstream target genes including cell death regulator p53. GAPDH = glyceraldehyde-3-phosphate dehydrogenase; GOSPEL = GAPDH’s competitor of Siah Protein Enhances Life; Siah1 = Seven in absentia homolog; GSH = glutathione; H_2_O_2_ = hydrogen peroxide.

Moreover, if there is too much oxidative (specifically nitrosative) stress, *S*-sulphenylated GAPDH is not *S*-glutathionylated, but *S*-sulfinated (Figure 2). Instead of a redirection of the metabolic flux to the pentose phosphate pathway, GAPDH does not complex with GAPDH’s competitor of Siah protein enhances life (GOSPEL) protein, but with seven in absentia homologs (Siah1), which has nuclear localization signal [18]. Nuclear GAPDH is acetylated by the histone acetyltransferase CBP/p300. Acetylated GAPDH subsequently stimulates the acetylating function of CBP/p300. Consequently, p300 is activated and is able to activate its downstream targets including the tumor suppressor gene p53 [27]. p53 is known to cause cellular dysfunction and cell death [17]. However, this kind of adaptation should be seen on cellular level, indicating that cells that have too much stress are not able to repair the damage induced. For a tissue it could be advantageous that these cells induce their own programmed cell death before they change into dangerous mutated cells. 

### 3.2. Epigenetic Level

Oxidative stress has been shown to have an effect on epigenetic processes. ROS can result in an oxidation of 5-methylcytosine to 5-hydroxymethylcytosine [28,29]. Additionally, Jumonji C (JmjC) histone lysine demethylases and ten-eleven translocation (TET) DNA hydroxylases are regulated by ROS because their ferrous catalytic center is inhibited by oxidation to the ferric state [28,30,31]. If the ratio of NAD^+^/NADH is increased, this affects the activity of class III histone deacetylases (HDACs) [28,30,31]. An adaptive increase in HDAC activity is expected. Additionally, NAD^+^ also increases histone deacetylases of the sirtuin family, SIRT1-3 and SIRT6 [32]. Due to the low K_m_ values of histone deacetylases for several physiological acyl substrates, NAD^+^ levels control histone deacetylase activity.

The effect of ROS on epigenetic processes could also lead to altered GSH levels. The targeting of histone demethylases, like Lsd1 by ROS, can induce an epigenetic adaptation of GSH levels. In diabetic retinopathy, Lsd1 inhibition prevented glucose to induce a decrease in H3K4me1 at the promotor of the Nrf2-regulated Gclc gene, which codes for the catalytic subunit of γGCS [33]. An increase in histone methylation at this site increased GSH synthesis, thereby preventing further development of diabetic retinopathy [33]. 

Interestingly, GSH also appears to have an effect on the regulation of epigenetic processes; for example, GSH reduces the activity of DNA methyltransferases (DNMTs) and histone methyltransferases (HMTs) by an inhibition of *S*-adenosyl methionine synthetase MAT1A, which provides the endogenous methyl-donor compound *S*-adenosyl methionine (SAM) [30]. In line with this, addition of *N*-acetylcysteine seems to decrease global DNA methylation and expression of DNA methyltransferases DNMT1 and DNMT3b [34].

Finally, epigenetic changes can be inherited over generations in humans, indicating that if a mother is exposed to a high level of oxygen that induced oxidative stress and resulted in epigenetic changes, thereby up-regulating the GSH system epigenetically, can also be transmitted to the child [35]. This is an adaptive mechanism that prepares the child for future exposure and ensures that its ability to survive is high in the future expected environment. Therefore, these transgenerational changes in gene expression contribute to the development of species and evolution. 

### 3.3. Genomic Level

Finally, the last long-term adaptive response can be found in the genome. Changes in the genome contribute to the development of species and evolution. Only if a species is adapted to the environment, will it survive. The fact that only phototrophic eubacteria contain GSH indicates that GSH evolved in parallel to oxygenic photosynthesis [36]. Furthermore, GSTs are evolutionarily-conserved, and homologous domains can be found back to early prokaryotes. The GSTT class has the most sequence identity with early GSTs, whereas GSTA, GSTM, GSTP forms are only found after gene duplication in animals and fungi [37]. In bacteria and plants, GSTs mainly function as stress-related/stress-resistant proteins [38]. However, over time, when oxygen appeared in the atmosphere, GSTs evolved from early stress-related/stress-resistant proteins to a protection mechanism against oxygen toxicity. Today, GSTs function in mammals as part of the phase 2 detoxification system in the liver. They are coupled to cytochrome P450 enzymes that mainly oxidize xenobiotics that are subsequently conjugated with GSH to facilitate excretion by the kidneys by increasing its water solubility [39]. However, it should be kept in mind that this metabolism does not always lead to detoxification, but could also be involved in bioactivation [39]. All in all, this shows how different parts of the GSH system evolved over time due to the requirements of the changing level of O_2_ in the environment. Remarkably, changes on the epigenetic and genomic level are more persistent. This means that it is present in an individual and does not easily change over a lifetime. For the individual, it is almost not possible to induce adaptation on this level, but on the level of the species this can be seen as an adaptive response since it ensures the survival of a complete species—the highest outcome of adaptation.

## 4. Factors Influencing Hormesis

Several factors are involved in inducing an adaptive response and restoring homeostasis. The nature of the induced hormetic response is dependent on the dose, the reactivity of the compound involved, and the timing of exposure (Figure 1). 

## 5. Dose and Reactivity of the Compound and Hormesis

First, the dose of the compound should be high enough to induce a significant adaptive response. However, if the dose is too high, the toxicity of the chemical will overrule and no adaptive response will occur.

Additionally, the compound’s reactivity is also crucial, since some compounds are more potent at a lower dose than others in inducing hormesis via a specific pathway. For example auranofin is more potent in inducing adaptation via Nrf2 than sulphoraphane [40]. For each compound the borders between non-toxic, sub-toxic, and toxic can be different (Figure 1). Additionally, the non-toxic level could also be found at another height in the coordinate system; for example, for O_2_, the non-toxic level is defined to be around 21% of the atmosphere, whereas for acrolein the World Health Organization recommends keeping the exposure as low as reasonably achievable (ALARA), but at least below the lowest observed adverse effect level (LOAEL) of 0.16 mg/m^3^. Additionally, the effect of the α,β-unsaturated aldehyde acrolein, a compound released during smoking or formed during incomplete combustion, on hormesis and adaptive responses is determined.

## 6. Adaptation Induced by Acrolein

### 6.1. Short-Term—Enzymatic Level

An important detoxification mechanism to protect against the damage of the electrophile acrolein is conjugation with GSH by glutathione-*S*-transferases (GSTs) in the liver [41,42,43]. GSTs are present in the cytosol, mitochondria, and microsomes. In humans, seven different cytosolic GST classes exist. The microsomal GSTs are membrane-bound GSTs and in humans three different isotypes exist that belong to MAPEG (Membrane Associated Proteins in Eicosanoid and Glutathione metabolism) [42,43]. GSTs are redox-sensitive enzymes that can be modified at their sulfhydryl groups. Xenobiotics like acrolein react with MGST1, a membrane-bound GST, and can increase MGST1 activity both in vitro and in vivo [12,44]. This indicates that the more acrolein is present forming adducts with sulfhydryl groups in MGST1, the more the conjugating activity of GST is stimulated, and the more protection is induced [12]. This is a form of short-term redox adaptation to electrophilic xenobiotics that modify a redox-sensitive enzyme and thereby increase cellular protection against such damaging compounds.

Acrolein also reacts with GSH and induces a short-term redox adaptation of the activity of γGCS, the rate-limiting enzyme of GSH synthesis. Acrolein stimulates γGCS activity and associated GSH synthesis by two mechanisms. Firstly, it oxidizes the disulfide bond between the regulatory and catalytic subunit of γGCS. This induces a conformational change of the enzyme which increases the affinity of the catalytic subunit for its substrates [15]. Secondly, it reduces cellular GSH levels resulting in an enhanced transcription of γGCS’ catalytic subunit [16]. The last mechanism is defined as long-term adaptation and will be explained in further detail in the next paragraph. Eventually, both mechanisms contribute to an enhanced GSH synthesis in cells exposed to acrolein. 

### 6.2. Long-Term Adaptation

#### 6.2.1. Transcriptional Level

As an electrophile, acrolein can also induce adaptation within hours to days by up-regulating the transcription of endogenous antioxidants. On a molecular level it was shown that acrolein adducts Keap1, thereby increasing nuclear translocation of Nrf2 and enhancing transcription of ARE containing antioxidant genes, including γGCS [45]. This subsequently contributes to an increased GSH synthesis and increased protection against these damaging electrophiles on a longer term. 

#### 6.2.2. Epigenetic Level

In addition, acrolein has been shown to decrease the activity of HDACs, because it adducts thiols in this enzyme. Since HDACs, including sirtuins, are inhibited, lysine residues on histones are not deacetylated anymore and this prevents HDACs from counteracting the expression of specific genes, possibly including the rate-limiting enzyme of GSH synthesis, γGCS [28]. In the end this will result in an up-regulation of γGCS. Additionally, acrolein is thought to activate p38MAPK that have been shown to have an effect on the activity and expression of certain lysine acetyl-transferases (e.g., KAT3A/KAT3B (CBP/p300)) [36,46]. Furthermore, via this mechanism, the expression of GSH system-related genes could be modified. 

### 6.3. Adaptation Induced by One Compound Protects against Another

Additionally, some compounds can induce an adaptation that provides protection against the toxic effects of another compound [47]. This phenomenon is called crosshormesis. This phenomenon implies that exposure to a non-toxic dose of a specific compound induces protection against another compound. For example hydrogen peroxide pre-treatment with a low dose has been shown to protect against acetate, ethanol, and propionate [48]. If the pretreatment is done with a compound that is generally defined as healthy, then crosshormesis is referred to as para-hormesis [49]. For example, relatively nontoxic nutritional antioxidants, like flavonoids, can up-regulate the endogenous antioxidant response during oxidative stress thereby protecting against the damaging effects of electrophiles [50]. From a toxicological point of view, both processes are the same because Paracelsus’s principle indicates that if the dose is high enough any compound can be toxic. 

### 6.4. Adaptation Induced by Dietary Antioxidants

**Figure 3:**
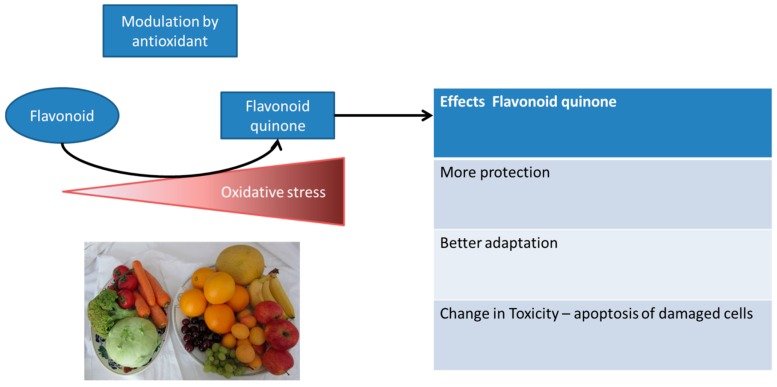
Example of exposure with a flavonoid that only induces different levels of redox adaptation in time in its oxidized form (flavonoid quinone). The more oxidative stress that is present, the more flavonoid scavenges the present ROS and the more flavonoid quinone is formed.

Additionally, next to the societal interpretation of flavonoids being healthy, these flavonoids compared to O_2_ and acrolein also show another level of hormesis induction, which makes it very interesting to further examine the hormetic mechanism indirectly induced by flavonoids. Next to the finding that other compounds can induce a similar mechanism of hormesis, the flavonoid on its own does not induce hormesis. However, if the cell experiences an increased level of oxidative stress, a flavonoid functions as a direct antioxidant, scavenging any damaging ROS (Figure 3). During this scavenging flavonoids are oxidized forming a flavonoid quinone that selectively channels the reactivity in the endogenous antioxidant system by reacting with thiols like GSH. If the level of stress increases even more, thiol-containing proteins are also targeted; with regard to flavonoids, this could be Keap1, as mentioned earlier in this article [50]. This results in increased expression of Nrf2-mediated antioxidant genes and increases protection. However, if the level of stress is even higher and the time of exposure is increased, even more thiol-containing proteins are attacked, like Ca^2+^-ATPase and GAPDH, which could selectively induce toxicity by inducing apoptosis [51,52]. 

## 7. Effects of Hormesis—Remodeling of the GSH System 

Adaptation processes over time have physiological consequences. For example, in the epithelial lining fluid of chronic smokers, who are regularly exposed to acrolein, the GSH levels are increased two-fold compared to non-smokers or acute smokers [13]. This suggests that inhaled cigarette smoke compounds probably induce an adaptive response of the protecting endogenous antioxidant system on a long term. Additionally, current smokers and ex-smokers had an increased DNA methylation level at the promotor of γGCS. This epigenetic modification probably contributes to the increased level of GSH in the epithelial lining fluid of these smokers [53]. 

Additionally, multiple studies show that athletes who experience a burst in oxidative stress during training have a higher antioxidant capacity than their sedentary controls, independently of their antioxidant intake [54]. Antioxidant capacity was determined as trolox-equivalent antioxidant capacity (TEAC) and activity of the antioxidant enzymes superoxide dismutase, glutathione peroxidase, and glutathione reductase were measured, in addition to α-tocopherol [54,55]. Except for α-tocopherol, all plasma concentrations of antioxidant parameters in athletes were above resting values and remained high for a longer time after exercise compared to sedentary subjects [55]. Furthermore, oxidative stress markers were measured, including malondialdehyde, representing the degree of lipid peroxidation [55]. Exercise is accompanied with an increase in oxidative stress, which finally results in adaptive changes in the (endogenous) antioxidant levels. Additionally, in endurance runners a negative association between total antioxidant status and protein carbonyl content, measuring oxidative damage towards proteins, was seen [56]. Moreover, when comparing the malondialdehyde and protein carbonyl levels in untrained, aerobically trained, and anaerobically trained individuals before and after exercise, remarkably only in untrained individuals the malondialdehyde and protein carbonyl level increased, whereas total antioxidant levels did not change [57]. Furthermore, depending on the type—endurance or resistance exercise—exercise also induces adaptive changes in muscle fiber types, causes muscle hypertrophy, induces metabolic adaptation by increasing size and number of mitochondria, and changes the activity of oxidative enzymes. These adaptations have been shown to result in long-term cardiac and respiratory changes by increasing cardiac output and stroke volume and stimulation of capillary formation. This, and the higher pulmonary blood flow, keep blood pressure stable even when changing the exercise level by increasing tidal volume, respiratory rate, and pulmonary diffusion [58]. Apart from the centrally-found physiological changes, also on the organ level (for example in the central nervous system and in (skeletal) muscles), physiological indications of redox-related adaptive responses could be found after exercise-based regimes [59,60,61,62]. When comparing the antioxidant levels, protein carbonyl content, and lipid peroxidation degradation products in the brain cortex and hippocampus of exercise- and non-exercise-exposed female CD1 mice it was found that only in exercise-exposed mice the antioxidant levels increased over time, whereas the protein carbonyl content and lipid peroxidation degradation products decreased [59,60]. Regular exercise or training has been shown to induce ROS-mediated adaptive responses in the skeletal muscles, but also in the heart and, finally, result in increased expression and level of endogenous antioxidant enzymes [61]. Remarkably, in the different organs the exercise-induced adaptive response seems rather similar, despite the fact that skeletal muscle, liver, and brain have different metabolic rates and functions during exercise [62]. The overall effect of exercise in the different organs is less oxidative damage, an increased level of endogenous antioxidant systems, repair processes, and a better protection against a future challenge with oxidative stress. 

Next to exercise, caloric restriction has also shown to have physiological effects due to adaptation. The harsher the caloric restriction in rats, the higher the level of malondialdehyde and the lower the level of glutathione and antioxidant enzymes, including superoxide dismutase [63]. This implicates that fasting or caloric restriction apparently triggers crucial hormetic improvements in the redox biology of cells and tissues.

Moreover, another example of the physiological consequence of adaptation is the effect of radiation on the human body. For a long time the linear no threshold model was applied, indicating that the more a subject is exposed to radiation, the higher the risk becomes to develop cancer. However, recently more evidence has been found to support a phenomenon called radiation hormesis. Repeated low dose radiation appears to induce adaptive mechanisms that result in protection and reduces the risk to develop cancer [64]. A meta-analysis of 18 studies performed between 1986 and 2012 showed that in mice doses between 0–100 mGy results in protection, whereas above 100 mGy the probability to induce damage is higher than the possibility of damage reduction [65]. Furthermore, in medical professionals working with radiation before 1950, a high death rate due to leukemia was seen, while after 1950, when the maximal exposure limit was reduced from 30,000 mSv/year to 50 mSv/year, medical professionals working with radiation have lower mortality rates than other medical specialists not working with radiation [66]. Finally, also in Chernobyl birds, at first GSH levels decreased and oxidative damage was increased, but over time, depending on the level of background radiation, oxidative stress and DNA damage showed a reduction, while an increase in GSH levels and body condition was observed, indicating that over time organisms can adapt to their exposure [67]. The mechanisms, by which radiation induces adaptation, are largely unknown, but certain findings underline that the immune system is boosted by low dose radiation, DNA repair is enhanced, and endogenous antioxidants are increased, probably by the activation of the Nrf2 pathway [66]. 

### Timing and Hormesis

**Figure 4:**
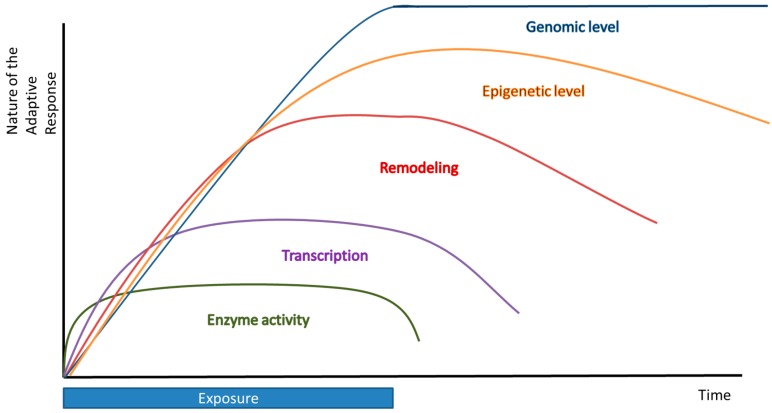
Multiple levels of redox adaptation over time.

Finally, timing is very essential for hormesis to take place. The timing between the first and second exposure is critical in the fact that if these exposures follow each other too rapidly, there is not enough time for the induction of a protective adaptive response and the second treatment might be lethal. Moreover, if the time between these exposures is too long, the adaptive effect will fade away and no effect of the first treatment will be seen. The timing between two exposures is also compound-dependent. Furthermore, it would be very interesting to elucidate the dynamics of this adaptation process. Could cells or organisms exposed repeatedly to a low dose of a compound continuously lead to a persistent adaptive response or will the effect of this fade out, causing a kind of desensitization? Additionally, the duration of the adaptive effect is another factor involved in adaptation (Figure 4). We distinguish short-term and long-term adaptation. Short-term adaptation can be defined as a direct adaptation carried out in a few seconds or minutes; for example, an enzymatic regulation. Long-term adaptation includes an adaptation from which the effects can even be noticed after hours or days (transcriptional regulation), months or years, or even generations (epigenetic and genomic effects). 

## 8. Conclusions

In conclusion, changes in the environment will induce adaptive mechanisms that mitigate or eventually counteract the change. For example, adaptation ensures that an increase in oxygen pressure is not lethal by upgrading of the antioxidant defense (Figure 1). Chronic changes of the environment may lead to a new steady state. As illustrated in Table 2, the longer the exposure time, the more persistent the adaptation becomes, or the organism cannot cope with the exposure any longer and dies. The most drastic fatal adaptation is seen at the genome level, which may even lead to new species. In contrast, adaptation at the functional protein level is characterized by more direct, temporary, and subtle changes (Table 2). Thus, multiple levels of redox adaptation exist in time, which differ in time of onset and duration. 

Short-term adaptation includes direct enzymatic modification, or a change in the available amount of cofactors, or an alteration of signaling molecules in a specific pathway which will result in an effect in seconds or minutes. Redox regulation on a transcriptional level generally takes hours or days. These relatively short-term adaptive mechanisms belong to the phenomenon called hormesis. In addition, adaptation can develop on a time scale of months or years. These are not covered by the term hormesis, but can be seen as a kind of adaptation. This includes remodeling of the GSH system, and epigenetic and genomic effects. Remodeling could lead to beneficial (increase in antioxidant capacity after exercise) or deleterious (apoptosis) consequences and result in another phenotype. The epigenetic changes have more persistent adaptive effects that may last over generations. The most persistent and drastic form of adaptation is seen when the genome is adjusted. In the current paradigm a random mutation of DNA may lead to species that better fit in an environment that has changed. 

It should be realized that, ultimately, all forms of adaptation are encoded in the genome are universal among living organisms, including the presence of redox-sensitive thiol groups in the microsomal GST and Keap1. Based on the aforementioned paradigm that in the genome only information that fits is preserved, it might be reasoned that the ability of an organism to give a hormetic response is beneficial on an evolutionary level. Despite the energy the cell has to invest in a hormetic response, this, as also discussed in this review, might come too early or too late, and might also be unnecessary because the change in the environment was only temporary. This might be a problem for an individual (since in those cases it will not survive), but in the long-term those “investments” (of individuals) are vital/crucial for life on earth to survive and evolve, and in this way adapt to the ever-changing environment. 

In summary, many physiological processes follow a hormetic response and several factors are involved in inducing an adaptive response. When investigating a biological response, adaptive mechanisms should be taken into account considering not only the dose or the compound, but also time. Advanced knowledge about the hormetic responses will open new avenues to promote disease interventions aimed at increasing our ability to adapt. Increasing our ability to adapt can be used to cope with changes in the environment, like oxidative stress, that otherwise would result in diseases like cardiovascular disease, cancer and metabolic diseases.

## Figures and Tables

**Table 1:** The short term adaptive mechanisms. GSH = glutathione; O_2_ = oxygen; γGCS = γ-glutamyl-cysteine synthetase; GR = glutathione reductase; NADPH = Nicotinamide adenine dinucleotide phosphate.

Short Term GSH-Related Adaptation to O_2_	Target
Cofactor	GSH
Enzyme	γGCS
Regeneration	GR- > NAPDH

**Table 2:** The effect of time and frequency of exposure on adaptive changes. If the environment changes (for example, the O_2_ pressure), the steady state will be restored directly; in the long-term the steady state will be adapted.

Exposure	Time	Frequency	Adaptation	Example	Effect	Fading Effect	Outcome	Change	
**Single exposure of relative low dose**	**Direct/acute**	**Occasionally**	**Enzymatic activity**	**GSTs**	**Fast**	**Fast**	**Hormetic Response**	**Protein**	**Reversible**
			More cofactors	GSH synthesis GAPDH					
		**Often**	**More transcription**	**Nrf2**			**Altered Phenotype**		
			Remodeling		Slow	Not			
**Chronic exposure of relative high dose**	**Long**	**Continuously**	**Epigenetic level**				**Altered Genotype**		
			Genomic level					**Gene**	**Irreversible**
